# Supplementary material for: Antibiotic—Lysobacter enzymogenes proteases combination as a novel virulence attenuating therapy
Source: PLoS One. 2023 Mar 9;18(3):e0282705. doi: 10.1371/journal.pone.0282705 (PMC9997937; doi:10.1371/journal.pone.0282705)
Supplement: S2 Fig — (DOCX) [file pone.0282705.s002.docx]

**Antibiotic – Lysobacter enzymogenes proteases combination as a novel virulence attenuating therapy**

Ghadeer A.R.Y. Suaifan^1^*^¶^, Diana M.A. Abdel Rahman^1¶^, Ala’ M. Abu-Odeh^2&^, Fahid Abu Jbara^3&^ Mayadah B. Shehadeh^1¶,^ Rula M. Darwish^3¶^

1 Department of Pharmaceutical Sciences, School of Pharmacy, The University of Jordan, Amman, Jordan

^2^ Department of Pharmaceutical Chemistry and Pharmacognosy, School of Pharmacy, Applied Science Private University, Jordan, Amman.

^3^ School of Medicine, The University of Jordan, Amman, Jordan

^4^ Department of Pharmaceutics and Pharmaceutical Biotechnology, School of Pharmacy, The University of Jordan, Amman, Jordan

*Corresponding author

E-mail: gh.suaifan@ju.edu.jo (GARYS)

E-mail: Ghadeer_petra@yahoo.com (GARYS)

¶ These authors contributed equally to this work.

& These authors also contributed equally to this work.

| **I** | 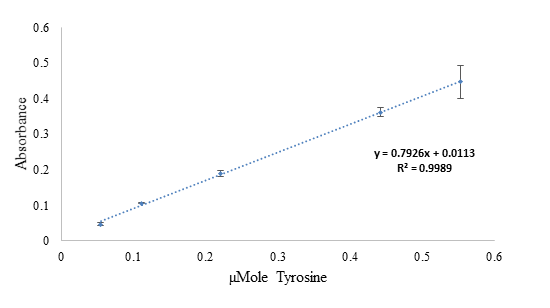 |
| --- | --- |
| **II** | 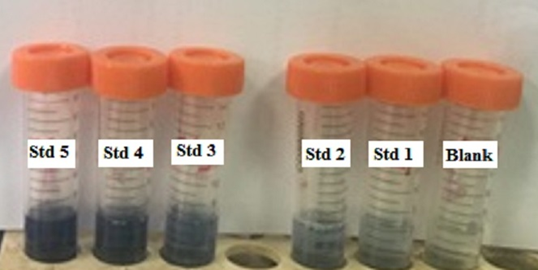 |
| **Fig. S2: Sigma's non-specific protease activity assay.** **I:** *L*-tyrosine standard curve;  **II:** *L*-tyrosine standard stock solutions. | |
