# Supplementary material for: Antibiotic—Lysobacter enzymogenes proteases combination as a novel virulence attenuating therapy
Source: PLoS One. 2023 Mar 9;18(3):e0282705. doi: 10.1371/journal.pone.0282705 (PMC9997937; doi:10.1371/journal.pone.0282705)
Supplement: Attachment — Submitted filename: Suaifan et al. response to reviewers.docx [file pone.0282705.s003.docx]

Dear Editor,

Thanks for your letter sent on May 6^th,^ 2022 regarding our manuscript Ref. No.:  PONE-D-22-05831, entitled: " **Antibiotic – *Lysobacter enzymogenes* proteases combination as a novel virulence attenuating therapy**". Please consider our revised manuscript entitled: which has been amended based on reviewer comments to meet PLOS ONE criteria for publication.

**Reviewers' comments:**
Reviewer #1: The authors have tried to study the interaction between *Lysobacter enzymogenes* proteases and antibiotics in the manuscript entitled “Antibiotic – *Lysobacter enzymogenes* proteases combination as a novel virulence attenuating therapy”.

Response: Thanks for the reviewer’s interesting comments.

*****However the value of this research and its importance to the scientific community is questionable if the *L. enzymogenes* proteases has inhibitory activity (toxicity) towards mammalian cells.

A) A new experiment has been conducted to resolve reviewer concern about the value of this research and its importance to the scientific community in relation to *L. enzymogenes* proteases toxicity towards mammalian cells

*** *Cytotoxic Activity***

**"** MTT assay was applied to evaluate *L. enzymogenes* CFS cytotoxicity. The cell line used in this assay was human normal skin fibroblast (CCD-1064SK) purchased from the American Type Culture Collection (ATCC, Manassas, VA, USA). This colorimetric assay measures the cellular metabolic activity based on the ability of nicotinamide adenine dinucleotide phosphate (NADPH)-dependent cellular oxidoreductase enzymes to reduce the yellow 3-(4,5-dimethylthiazol-2yl)-2,5-diphenyltetrazolium bromide dye ( MTT) to form the insoluble formazan purple crystals [48-50]. Cells were seeded in a 96-well culture plates in a final volume of 100 µL media per well, then plates were incubated in a humidified atmosphere (37°C, 5% CO2) for 24 h to allow cells to adhere. When cells reached confluency, they were treated with the selected crude to obtain final concentrations of 1.25, 2.5, 5, 10, 20, 25, 40, and 50 % (v/v). Cells were incubated in the humidified atmosphere (37°C, 5% CO2) for 72 h before performing the MTT assay to determine the cells’ viability. Experiment was conducted in triplicate, and the control cells were treated only with 10 % TSB. The optical density for the treated and control wells was measured at 570 nm using a microtiter plate reader (BioTek, Winooski, VT, USA). Percentage viability was calculated using the formula below

% Viability =((Mean OD sample)/(Mean OD blank))×100**"	Page 9, Line 207-223.**

* Fig. 5 in the revised version presents no toxicity for *L. enzymogenes* CFS at tested concentrations.

**Fig. 5** Viability of human normal skin fibroblast (CCD-1064SK) at different concentrations (1.25, 2.5, 5, 10, 20, 25, 40, and 50 % (v/v)) of *L. enzymogenes* CFS as assessed by MTT assay. **Page 16, Line 321.**

* Antimicrobial peptides are often limited by their cytotoxicity[59]. The *in vitro* cytotoxicity is quantitatively evaluated by MTT assay. This bioassay is based on the intracellular reduction of methyltetrazolium salt by the viable cells[60, 61]. According to the International Organization for Standardization (ISO) 10993-5 [62], tested materials that reveal a reduction in cell viability by more than 30% are regarded as cytotoxic. The present research indicated high cell viability above 90% for all *L. enzymogenes* CFS tested concentrations. **Page 18, Line 368-373.**

*****After careful evaluation of the manuscript, I think the manuscript is currently not of publication standard and requires a significant rewrite/modification to bring it in-line with the standards of academic scientific publications.

Response: The revised version sections [Abstract, Introduction, Results, Discussion and Conclusion] have been rewritten to bring the article in-line with the standards of academic scientific publications.

***** The introduction does not adequately provide the background information on the antimicrobials discussed and the aims and objectives of the paper are not clearly defined.

Response: Introduction has been rewritten to adequately provide the background information regarding *Lysobacter* genus and its important role in controlling pathogen-induced diseases. **Page 3, Line 63-88.**

*****The methods section needs to include more details to ensure reproducibility.

Response: Method section has been amended. A minimum of three trials for each test was performed. Error bars have been added to the figures in the revised version for the average mean of three readings.

**Page 5, Line 121-124.**

**Page 7, Line 153-160.**

**Page 7, Line 177-180.**

**Page 7, Line 185-205.**

*****Additional statistical analysis is required to show statistical significance of all the methods undertaken and additional controls of L. enzymogenes proteases is needed, apart from the assays that are currently included.

Response: A new subsection entitled (***Statistical Analysis***) has been added to Material and Method section. **Page 9, Line 224-227.**

Also, statistically significant results were shown in **Fig**. 4 in the revised version. **Page 14**

Negative control was always tested along with samples and used in the calculation of the % growth inhibition as shown in the equation below

$$\% Growth Inhibition=100-\left( \frac{\mathrm{OD}T-\mathrm{OD}\mathrm{TB}}{\mathrm{OD}FG-\mathrm{OD}\mathrm{FB}} \right)\times100\%$$

Where:

-**OD_T_** is the average optical density of three replicates at 600nm for the tested target (*L. enzymogenes* *CFS* or antibiotics or the combination)

**-OD_TB_** is the average optical density of three replicates at 600nm for the tested target blank (10 μL of pathogen is replaced by equivolume of the appropriate sterile broth)

-**OD_FG_** is the average optical density of three replicates at 600nm for the study pathogen full growth.

**-OD_FGB_** is the average optical density of three replicates at 600nm for the study pathogen blank (No pathogen, only 10 μL of broth is used). **Page 8, Line 197.**

***** The results need to be elaborated properly, I think the data is presented poorly and the discussion is not fully supported by the results.

Response: Results section in the revised version was rewritten and new figures were added to display the study results and support the discussion section. **Page 11, Line 251. Page 12, Line 274.**

*****In the discussion section, sentences are too long, that makes it difficult to understand. Sentences should be clear and short.

Response: Discussion section was revised to sort out this point.

*****The whole manuscript would benefit from proof reading by some peers of the field in order to bring it in line with the standards required for academic publication style. I think the authors did not put much efforts and attention while writing the manuscript and preparing the figures.

Response: The revised version has been proof read.

***** Overall I believe, L. enzymogenes proteases cannot be considered as good antibacterial agents with 16% maximum growth inhibition.

Response: Thanks for the reviewer comment. The aim of this study was to investigate the implication of *Lysobacter enzymogenes* bioactive proteases alone and in combination with antibiotics available in the market to combat bacterial resistances. The potential restores of cefixime inhibitory activity against MRSA when combined the environmentally friendly *L. enzymogenes* represent an efficient tool in the battle against multidrug-resistant pathogens. However, further studies are required to understand the exact mechanism.

**Reviewer #2**

The major points that need to be addressed are mentioned below:
1. There is a lot of grammatical, spelling, and punctuation mistakes. Please check your manuscript from a native English speaker or give it to a professional English editing services.

Response: The revised version has been proof read.

2. It is not clear why the authors have chosen *Lysobacter enzymogenes* proteases as a potentiator for the combination study. Have you screened this among other proteases? If so, please include them. If not, then please provide some background information in the introduction section whether such kind of study have been done before or not. The rational or approach looks unclear.

Response: Thanks for the reviewer. The introduction has been rewritten to provide essential background information which support the aim of this study. **Page 3-4, Line 63-88.**

3. In the last section of introduction, the authors have mentioned that they have analysed α and β- lytic proteases in the study, however there is no mention of the types of the proteases thereafter in the entire text.

Response: Thanks for the reviewer. This study aims to investigate *L. enzymogenes* cell free supernatants bioactive crude proteases. Further studies to be carried in the future are essential to identify the specific protease capable of potentially inhibit bacterial growth.

4. The methods should provide enough detail to allow others to repeat the work, e.g range of the concentrations used.

Response: Method section has been amended. A minimum of three trials for each test was performed. Error bars have been added to the figures in the revised version for the average mean of three readings.

**Page 5, Line 121-124.**

**Page 7, Line 153-160.**

**Page 7, Line 177-180.**

**Page 7, Line 185-205.**

5. E.coli 31446 was not mentioned in the “Bacterial strains” section in material & methods.

Response: Thanks for the reviewer. This is a mistake. Only *E. coli* O157:H7 pathogen was included in the study.

6. Please cite the content of the sections such as “Preparation of L. enzymogenes culture supernatant proteases” and Quantitative evaluation of L. enzymogenes culture supernatant proteases antibacterial activity” in material & methods.

Response: Thanks for the reviewer note. Material used in the preparation of *L. enzymogenes* culture and in the measurement of the proteolytic activity was added to the Materials section.

**Page 4, Line 101-103.**

**Page 5, Line 113-116.**

7. How did the authors calculate number of bacterial cells in the % growth inhibition formula? Why E.coli 31446 was not used for this study?

Response: Thanks for the reviewer. In this study, antibacterial activity was expressed as percentage inhibition of bacterial growth following 24 h incubation at 37 °C and calculated using the following equation:

$$\% Growth Inhibition=100-\left( \frac{\mathrm{OD}T-\mathrm{OD}\mathrm{TB}}{\mathrm{OD}FG-\mathrm{OD}\mathrm{FB}} \right)\times100\%$$

Where:

-ODT is the average optical density of three replicates at 600nm for the tested target (*L. enzymogenes* CFS or antibiotics or the combination)

-ODTB is the average optical density of three replicates at 600nm for the tested target blank (10 μL of the pathogen is replaced by equivolume of the appropriate sterile broth)

-ODFG is the average optical density of three replicates at 600nm for the study pathogen full growth.

-ODFGB is the average optical density of three replicates at 600nm for the study pathogen blank (No pathogen, only 10 μL of broth was used).This is a mistake. Only *E. coli* O157:H7 pathogen was included in the study.

**Page 8, Line 197.**

8. Please maintain uniformity in writing the strains MSSA, MRSA. In some places, they are written with ATCC number and whereas in other places they are not.

Response: Thanks for the reviewer comment. Consistency was amended in the revised version.

9. As mentioned above, please cite from where the authors have adapted the experimental studies such as for broth microdilution minimum inhibitory concentration (MIC) test. Please mention in methods section “Determination of antibiotics minimum inhibitory concentrations”, whether the authors have studied MIC for MSSA.

Response: Thanks for the reviewer comments. Reference (46) was added to the experimental section and MIC procedure was clarified.

**Page 7, Line 153-160.**

10. In Table 1. Authors have mentioned the concentration of Levofloxacin hemihydrate and Gentamicin sulfate for the MIC testing. But if we go through the 2-fold serial dilution, the values which are mentioned in the results does not match with the dilution values. Table 1 concentration values does not match with the Table 2 result concentration values.

Response: Thanks for the reviewer comment. Table 1 was checked, and the serial dilution was clarified in the section. **Page 7, Line 156-160 and Line 164-167.**

11. The authors have not mentioned how statistical analysis was done, however they have shown statistical significance in the figures. It is missing in figure 4C though.

Response: A new subsection entitled (***Statistical Analysis***) has been added to Material and Method section. **Page 9, Line 224-227.**

Also, statistically significant results were shown in **Fig**. 4 in the revised version. **Page 14**

12. Please provide sub-headings for the result section.

Response: Thanks for the reviewer comments. Subsections were added in the revised version and highlighted in red.

13. In figure 1, please maintain the uniformity in font size and type. Legend is not properly visible in figure 1A.

Response: Thanks for the reviewer note. All figures in the revised version were checked for clarity and uniformity.

14. Line 181: Please mention the concentration of supernatant protease.

Response: Thanks for the reviewer. *L. enzymogenes* cell free supernatant tested in combination study was prepared from a bacterial cell culture (4% (v/v), i.e 0.5 ml of pure broth culture (10^7^ CFU/ml) with 12 ml broth) incubated for 11 days.

15. In figure 2, authors wrote E. coli 157:H7 in the legend, however it should be E. coli O157:H7.

Response: Thanks for the comment and apology for the mistake. Figure 2 is now presented as Fig. 3 in the revised version and has been amended.

16. In table 2, in the table title, authors wrote Levofloxacin hemihydrate and Gentamicin sulfate, while in the table, authors wrote only Levofloxacin and Gentamicin. Please maintain uniformity in Figure 4 and 5 as well.

Response: Thanks for the reviewer note. All antibiotics in the revised version were checked for uniformity.

17. In figure 3, 4 and 5 (A) the figure legends says something else, while the figure caption says something else. It is not clear whether the figure is for to MRSA or MSSA?

Response: Thanks for the reviewer note. Figure 3, 4 and 5 in the submitted version were replaced by Fig.4 in the revised article as requested by the first reviewer.

18. In figure 3A, if the authors have shown the figure for MRSA; how do they explain the MIC values in the table 2, where they have shown that Cefixime is not effective against MRSA. So, how did the authors consider 4ug/ml of Cefixime as 0.5MIC as indicated in the box in the figure?

Response: Thanks for the reviewer note. Figure 3, 4 and 5 in the submitted version were replaced by Fig.4 in the revised article as requested by the first reviewer. All data were checked. In the previously submitted version. Boxes highlighting the MIC values were shifted during pdf conversion.

19. Concentrations should be mentioned throughout results and figures.

Response: Thanks for the reviewer note. Concentration were mentioned throughout the article once needed.

20. The authors should check the cytotoxicity of the combination at various concentrations.

Response: Thanks for the reviewer comment:

B) A new experiment has been conducted to solve reviewer concern about the value of this research and its importance to the scientific community in relation to *L. enzymogenes* proteases toxicity towards mammalian cells

*** *Cytotoxic Activity***

**"** MTT assay was applied to evaluate L. enzymogenes CFS cytotoxicity. The cell line used in this assay was human normal skin fibroblast (CCD-1064SK) purchased from the American Type Culture Collection (ATCC, Manassas, VA, USA). This colorimetric assay measures the cellular metabolic activity based on the ability of nicotinamide adenine dinucleotide phosphate (NADPH)-dependent cellular oxidoreductase enzymes to reduce the yellow 3-(4,5-dimethylthiazol-2yl)-2,5-diphenyltetrazolium bromide dye ( MTT) to form the insoluble formazan purple crystals [48-50]. Cells were seeded in a 96-well culture plates in a final volume of 100 µL media per well, then plates were incubated in a humidified atmosphere (37°C, 5% CO2) for 24 h to allow cells to adhere. When cells reached confluency, they were treated with the selected crude to obtain final concentrations of 1.25, 2.5, 5, 10, 20, 25, 40, and 50 % (v/v). Cells were incubated in the humidified atmosphere (37°C, 5% CO2) for 72 h before performing the MTT assay to determine the cells’ viability. Experiment was conducted in triplicate, and the control cells were treated only with 10 % TSB. The optical density for the treated and control wells was measured at 570 nm using a microtiter plate reader (BioTek, Winooski, VT, USA). Percentage viability was calculated using the formula below

% Viability =((Mean OD sample)/(Mean OD blank))×100**"	Page 9, Line 207-223.**

* Fig. 5 in the revised version presents no toxicity for *L. enzymogenes* CFS at tested volume.

**Fig. 5** Viability of human normal skin fibroblast (CCD-1064SK) at different concentrations (1.25, 2.5, 5, 10, 20, 25, 40, and 50 % (v/v)) of *L. enzymogenes* CFS as assessed by MTT assay. **Page 16, Line 321**

* Antimicrobial peptides are often limited by their cytotoxicity[59]. The *in vitro* cytotoxicity is quantitatively evaluated by MTT assay. This bioassay is based on the intracellular reduction of methyltetrazolium salt by the viable cells[60, 61]. According to the International Organization for Standardization (ISO) 10993-5 [62], tested materials that reveal a reduction in cell viability by more than 30% are regarded as cytotoxic. The present research indicated high cell viability above 90% for all *L. enzymogenes* CFS tested concentrations. **Page 18, Line 368-373.**

21. Authors did not mention about the outcome of combination study whether the effect was additive, synergistic or antagonist?

Response: Thanks for the reviewer. At this level of research, we are testing the implication of *L. enzymogenes* cell free supernatant crude protease solution. It would be not possible to judge the exact effect as we are having crude protease solution. However, future research aiming at the isolation and identification of the proteases would facilitate the preparation of different concentrations, calculation of the MIC and determination of the exact effect. This of course needs a fund and would be considered in our future research.

22. In line 231: “As shown….in figure 1B, authors were talking about Figure 1A or 1B?

Response: Thanks for the reviewer note. It is Fig 1A. This mistake was taken into consideration in the revised version.

23. In line 266: “at half, quarter and eighth the MIC concentrations”. It should be written as 0.5, 0.25 and 0.125 MIC.

Response: Thanks for the reviewer comment. Sub MIC levels were formatted as requested by the reviewer in the revised version.

24. In the discussion section, authors did not discuss about the effect of L. enzymogenes proteases and antibiotics against E.coli strains, while it was described for Staphylococcus.

Response: Thanks for the reviewer comment. Discussion regarding L. enzymogenes growth inhibit activity against tested E. coli O157:H7 is described in the revised version.

**Page 12, Line 274-275**

**Page 14, Line 298-299**

**Page 17, Line 348-351**
