# Supplementary material for: Antibiotic—Lysobacter enzymogenes proteases combination as a novel virulence attenuating therapy
Source: PLoS One. 2023 Mar 9;18(3):e0282705. doi: 10.1371/journal.pone.0282705 (PMC9997937; doi:10.1371/journal.pone.0282705)
Supplement: Attachment — Submitted filename: Response to reviewers 2.docx [file pone.0282705.s004.docx]

Dear Editor,

Thanks for your letter sent on Dec 1^th,^ 2022 regarding our manuscript Ref. No.:  PONE-D-22-27664, entitled: " **Antibiotic – Lysobacter enzymogenes proteases combination as a novel virulence attenuating therapy**". Please consider our revised manuscript entitled: which has been amended based on reviewer comments to meet PLOS ONE criteria for publication.

1. Please ensure that your manuscript meets PLOS ONE's style requirements, including those for file naming. The PLOS ONE style templates can be found at

https://journals.plos.org/plosone/s/file?id=wjVg/PLOSOne_formatting_sample_main_body.pdf and

<https://journals.plos.org/plosone/s/file?id=ba62/PLOSOne_formatting_sample_title_authors_affiliations.pdf>

**Response: Thanks for the comment. We can ensure that revised version meets with PLOS ONE's style requirements.**

2. We note that the grant information you provided in the ‘Funding Information’ and ‘Financial Disclosure’ sections do not match.

When you resubmit, please ensure that you provide the correct grant numbers for the awards you received for your study in the ‘Funding Information’ section.

**Response:**

**Thanks for the comment. Funding Information and Financial disclosure sections in the current resubmission has been checked.**

3. Thank you for stating the following in the Acknowledgments Section of your manuscript:

"The authors (GS and MS) would like to acknowledge the Deanship of the Scientific Research at The University of Jordan (2073, 2460) and Hamdi Mango Center for Scientist Research (2017) for financial support. The funders had no role in study design, data collection and analysis, decision to publish, or preparation of the manuscript."

We note that you have provided funding information that is not currently declared in your Funding Statement. However, funding information should not appear in the Acknowledgments section or other areas of your manuscript. We will only publish funding information present in the Funding Statement section of the online submission form.

Please remove any funding-related text from the manuscript and let us know how you would like to update your Funding Statement. Currently, your Funding Statement reads as follows:

"The funders had no role in study design, data collection and analysis, decision to publish, or preparation of the manuscript"

Please include your amended statements within your cover letter; we will change the online submission form on your behalf.

**Response:**

1) **Funding-related text has been removed from the Acknowledgment section in the revised version.**
2) **Funding Statement " This research was supported by a grant (2073, 2460) from the Deanship of the Scientific Research at The University of Jordan and grant (2017) from Hamdi Mango Center for Scientist Research. The funders had no role in study design, data collection and analysis, decision to publish, or preparation of the manuscript. "**

4. Please include a copy of Table 2 which you refer to in your text on Response to Reviewers file.

**Response:**

**Apology as the article contains only table 1. There is no Table 2.**

5. Please review your reference list to ensure that it is complete and correct. If you have cited papers that have been retracted, please include the rationale for doing so in the manuscript text, or remove these references and replace them with relevant current references. Any changes to the reference list should be mentioned in the rebuttal letter that accompanies your revised manuscript. If you need to cite a retracted article, indicate the article’s retracted status in the References list and also include a citation and full reference for the retraction notice.

**Response:**

**Thanks for the comment. References were revised.**

1) **Reference number 7 in the submitted manuscript (Thesis) has been substituted by another article (Xu S, Zhang Z, Xie X, Shi Y, Chai A, Fan T, et al. Comparative genomics provides insights into the potential biocontrol mechanism of two Lysobacter enzymogenes strains with distinct antagonistic activities. Front Microbiol. 2022;13:966986.)**
2) **New references were added (Highlighted in red in the revised manuscript) to support the use of adjuvant therapy as a tool to unlock bacterial resistance.**

Additional Editor Comments (if provided):

The submitted MS has been revised accordingly. However, I have gone through the MS and found that some important earlies reports have not cited in the introduction and discussion sections of the paper. Therefore, Authors may cite the following references

RSC Adv., 2021, 11, 36459-36482; Microbial Pathogenesis (2022), 170, 105678; Bioorganic & Medicinal Chemistry Letters 27(18), 2017, 4221-4228; Trends in Biotechnology (2022); 40, 1004-1017; ACS Applied Nano Materials (2021); 4(2), 1512–1528; Scientific Reports, (2019); 9 6520; RSC Advances (2015), 5 (87), 71060-71070.

**Response:**

**Thanks for the editor comments. References below were added to the revised version (Disscussion section) to highlight the increased interest in adjuvants therapy to unlock bacterial resistance by different application involving natural substances and nanotechnology.**

**(51.	González-Bello C. Antibiotic adjuvants – A strategy to unlock bacterial resistance to antibiotics. Bioorganic & Medicinal Chemistry Letters. 2017;27(18):4221-8.**

**52.	Prateeksha P, Bajpai R, Rao CV, Upreti DK, Barik SK, Singh BN. Chrysophanol-Functionalized Silver Nanoparticles for Anti-Adhesive and Anti-Biofouling Coatings to Prevent Urinary Catheter-Associated Infections. ACS Applied Nano Materials. 2021;4(2):1512-28.**

**53.	Abd El-Aleam RH, George RF, Georgey HH, Abdel-Rahman HM. Bacterial virulence factors: a target for heterocyclic compounds to combat bacterial resistance. RSC Adv. 2021;11(58):36459-82.)**
